# Supplementary material for: Association of Maternal Longitudinal Hemoglobin with Small for Gestational Age during Pregnancy: A Prospective Cohort Study
Source: Nutrients. 2022 Mar 28;14(7):1403. doi: 10.3390/nu14071403 (PMC9003216; doi:10.3390/nu14071403)
Supplement: Supplementary file 1 [file nutrients-14-01403-s001.zip › nutrients-1648356-supplementary.pdf]

Supplement Table S1. Associations between Hb concentrations and SGA in middle and late pregnancy in women without gestational hypertension or preeclampsia.

Hb concentrations, g/L	n	SGA [n (%)]	Model 1† RR (95% CI)	Model 2‡ aRR (95% CI)	Model 3§ aRR (95% CI)
Mid-pregnancy	3057				
≥130	212	16 (7.6)	1.21 (0.72, 2.03)	1.41 (0.84, 2.36)	1.41 (0.84, 2.36)
120–129	941	62 (6.6)	1.06 (0.77, 1.45)	1.10 (0.80, 1.52)	1.10 (0.80, 1.52)
110–119	1298	81 (6.2)	Reference	Reference	Reference
<110	606	36 (5.9)	0.95 (0.65, 1.39)	0.86 (0.60, 1.25)	0.86 (0.60, 1.25)
Late pregnancy	3057				
≥130	358	41 (11.5)	2.01 (1.38, 2.93)	2.27 (1.55, 3.33)	2.29 (1.57, 3.36)*
120–129	790	57 (7.2)	1.27 (0.89, 1.80)	1.24 (0.88, 1.76)	1.25 (0.88, 1.77)
110–119	1052	60 (5.7)	Reference	Reference	Reference
<110	857	37 (4.3)	0.76 (0.51, 1.13)	0.69 (0.47, 1.03)	0.69 (0.47, 1.03)

Hb, hemoglobin; SGA, small for gestational age; RR, hazard ratio; CI, confidence interval; aRR, adjusted risk ratio.
†Model 1 unadjusted any covariates.
‡Model 2 adjusted for maternal age, ethnicity, education level, average personal income, pre-pregnancy BMI, active or passive smoking, alcohol consumption, parity, gestational weight gain and newborn gender.
§Model 3 included model 2 covariates and iron-containing supplement. *P<0.001

Supplement Table S2. Associations between longitudinal Hb change and SGA in women without gestational hypertension or preeclampsia, stratified by Hb in late pregnancy.

	n	Hb-middle	Hb-late	Iron supplement	SGA	Adjusted Model †
		Mean ± SD	Mean ± SD	[n (%)]	[n (%)]	Adjusted RR (95% CI)
Hb-late < 130						
Hb change < –6.0 g/L	885	120.2 ± 7.9	107.0 ± 8.9	442 (49.9)	30 (3.4)	0.55 (0.36, 0.84)*
Hb change –6.0 ~ 1.9 g/L	859	116.6 ± 7.7	114.2 ± 7.8	457 (53.2)	57 (6.6)	Reference
Hb change ≥ 2.0 g/L	955	110.8 ± 7.7	118.6 ± 7.3	524 (54.9)	67 (7.0)	1.09 (0.78, 1.53)
Hb-late ≥130						
Hb change < 8 g/L	109	130.3 ± 4.4	133.3 ± 2.5	66 (60.6)	11 (10.1)	0.84 (0.40, 1.77)
Hb change 8 ~ 15.9 g/L	127	123.0 ± 4.5	134.6 ± 4.2	85 (66.9)	16 (12.6)	Reference
Hb change ≥ 16 g/L	122	116.1 ± 6.7	138.8 ± 8.1	79 (64.8)	14 (11.5)	0.80 (0.41, 1.56)

Hb, hemoglobin; SGA, small for gestational age; RR, risk ratio; CI, confidence interval; aRR, adjusted risk ratio.

†Adjusted Model adjusted for maternal age, height, ethnicity, education level, average personal income, pre-pregnancy BMI, active or passive smoking, alcohol consumption, parity, gestational weight gain, newborn gender, iron-containing supplement, and Hb measurement interval.

*P<0.001
